# Maternal Malnutrition and Offspring Sex Determine Juvenile Obesity and Metabolic Disorders in a Swine Model of Leptin Resistance

**DOI:** 10.1371/journal.pone.0078424

**Published:** 2013-10-24

**Authors:** Alicia Barbero, Susana Astiz, Clemente J. Lopez-Bote, Maria L. Perez-Solana, Miriam Ayuso, Isabel Garcia-Real, Antonio Gonzalez-Bulnes

**Affiliations:** 1 UCM, Facultad de Veterinaria, Madrid, Spain; 2 INIA, Madrid, Spain; Wageningen University, Netherlands

## Abstract

The present study aimed to determine, in a swine model of leptin resistance, the effects of type and timing of maternal malnutrition on growth patterns, adiposity and metabolic features of the progeny when exposed to an obesogenic diet during their juvenile development and possible concomitant effects of the offspring sex. Thus, four groups were considered. A CONTROL group involved pigs born from sows fed with a diet fulfilling their daily maintenance requirements for pregnancy. The treated groups involved the progeny of females fed with the same diet but fulfilling either 160% or 50% of pregnancy requirements during the entire gestation (OVERFED and UNDERFED, respectively) or 100% of requirements until Day 35 of pregnancy and 50% of such amount from Day 36 onwards (LATE-UNDERFED). OVERFED and UNDERFED offspring were more prone to higher corpulence and fat deposition from early postnatal stages, during breast-feeding; adiposity increased significantly when exposed to obesogenic diets, especially in females. The effects of sex were even more remarkable in LATE-UNDERFED offspring, which had similar corpulence to CONTROL piglets; however, females showed a clear predisposition to obesity. Furthermore, the three groups of pigs with maternal malnutrition showed evidences of metabolic syndrome and, in the case of individuals born from OVERFED sows, even of insulin resistance and the prodrome of type-2 diabetes. These findings support the main role of early nutritional programming in the current rise of obesity and associated diseases in ethnics with leptin resistance.

## Introduction

Obesity and associated metabolic disorders are increasingly concerning issues. The World Health Organization (WHO) foresees that, by 2015, approximately 2.3 billion adults will be overweight and more than 700 million will be obese; the mortality rate due to diabetes will double between 2005 and 2030. The highest incidence of obesity and diabetes has been traditionally found in developed countries; currently, the incidence of these disorders is mainly increasing in rapidly developing regions, like China, India and countries of the Middle East. The situation is aggravated by a severe boost in the incidence of obesity at childhood in the last years. The number of overweight children under the age of five was over 42 million in 2010, according to WHO data; again, close to 35 million are living in developing countries.

Thus, there is an urgent necessity to tackle this health problem, by applying prevention strategies and focused treatments. Such actions need to be based on a thorough knowledge of obesity and its effects, by both observational and interventional research. However, interventional experimentation is not affordable in human beings and it needs to be performed on animal models. Most of the studies have been carried out in mice; however, the use of large animals (rabbit, sheep, pig) offers numerous profitable characteristics for translational studies. First, their body size allows application of the same imaging techniques routinely used in humans and serial sampling of large amounts of blood and tissues. Second, pathways regulating appetite, energy balance and adipogenesis are more similar to humans. Moreover, research in large animal species may provide valuable insights not only as a translational model for humans, but also directly for improving animal production, health and welfare. The most amenable large animal model (for proportional organ sizes, omnivorous habits, characteristics of lipoprotein metabolism and propensity to sedentary behavior and obesity is the pig [1-3].

A distinctive characteristic of people living in developing countries, besides ethnic differences, is their background of exposure to harsh environments and food scarcity and their current availability of nutrients in excess, mainly in the form of highly caloric obesogenic diets. There is a swine model, the Iberian pig [4,5], which is faced to similar conditions. The Iberian breed (*Sus scrofa meridionalis*) is genetically different from the modern commercial breeds (*Sus scrofa ferus*; [6,7]) and it has been reared in semi-feral harsh conditions for centuries. Thus, these animals have coped with seasonal cycles of feasting and famine by the strategy of storing excess fat during food abundance for surviving during food scarcity [8]. The Iberian pigs have a higher voluntary food intake and a higher trend towards adiposity than lean swine breeds and, when exposed to nutrients in excess, become obese [8,9] and even develop metabolic syndrome and diabetic prodrome [4].

Current theories support that obesity and associated disorders are the result of the interaction between genetic background and obesogenic environments, but strongly modulated by prenatal nutrition, either by excess or deficiency [10]. These hypotheses are summarized by the concept of the Developmental Origin of Health and Disease (DOHaD; [11]). The study of the DOHaD phenomena is mostly based on epidemiological studies in humans and on interventional studies in animal models. However, there are no previous studies assessing the interaction between prenatal nutrition and postnatal exposure to obesogenic diets in individuals genetically adapted, for generations, to harsh environments and food scarcity. 

Thus, the aim of the present experiment was to set a basis for such studies by determining the effects of maternal under- or overnutrition on growth patterns, adiposity and metabolic features of the progeny when exposed to an obesogenic diet during their juvenile development. A concurrent objective was to assess possible concomitant effects of the offspring sex. 

## Material and Methods

### Ethics statement

The study was performed according to the Spanish Policy for Animal Protection RD1201/05, which met the European Union Directive 86/609 about the protection of animals used in research. The experiment was specifically assessed and approved (report CEEA 2010/003) by the INIA Committee of Ethics in Animal Research, which is the named Institutional Animal Care and Use Committee (IACUC) for the INIA. 

### Animals and experimental design

The experiment involved a total of 69 Iberian pigs (Torbiscal strain) divided in four groups with different maternal nutrition level during pregnancy. A first group was the CONTROL group (n=10 males and 8 females), born from females fed with a standard grain-based diet (13.0% crude protein, 2.8% fat and 3.00 Mcal/kg of metabolizable energy) for fulfilling their daily maintenance requirements for pregnancy. Second and third groups were the progeny of females fed with the same diet but fulfilling either 160% (OVERFED group, n=9 males and 9 females) or 50% of daily maintenance requirements for pregnancy (UNDERFED group, n=6 males and 9 females) during the entire pregnancy; hence, preimplantational embryos and the subsequent fetuses of these both groups were exposed to prenatal programming. A fourth group was born from females fed with 100% maintenance requirements until Day 35 of pregnancy, like the CONTROL group, but restricted to 50% of such amount from Day 36 onwards, like the UNDERFED group (LATE-UNDERFED group, n=10 males and 8 females); hence, fetuses but not preimplantational embryos were exposed to prenatal programming in this group, by feed restriction during the last two thirds of pregnancy. In summary, a 4x2 factorial arrangement was used to study the effects of maternal nutrition and offspring sex on development and metabolic status of the progeny from birth to adulthood.

The assessment of the offspring development was carried out during the early postnatal (from birth to weaning at 28 days of age) and juvenile periods (from 28 to 240 days of age). Assessment of phenotypic characteristics at adulthood was done when the pigs reached adult body size and weight accordingly to breed-standards.

After weaning, at 28 days of age, all the piglets were housed, sorting out males and females, in collective pens at the INIA Animal Laboratory Unit (Madrid, Spain). At the first month after weaning, the piglets were fed with a standard diet with mean values of 18% of crude protein, 4.5% of fat and 3.35 Mcal⁄kg of metabolizable energy. Afterwards, from 60 to 120 days of age, the piglets were fed a diet containing mean values of 15.1% of crude protein, 2.8% of fat and 3.08 Mcal⁄kg of metabolizable energy; the amount of food offered was re-calculated with age for fulfilling daily maintenance requirements. From 120 days of age, for inducing the expression of obesity, the pigs had *ad libitum* access to the same diet but enriched in fat (6.3%) and, hence, with 3.36 Mcal/kg of metabolizable energy. 

### Evaluation of growth patterns and corpulence during juvenile development

In all the pigs, body weight and size (trunk length and abdominal and thoracic circumferences, correspondingly to AC and TC, obtained with a measuring tape) were recorded monthly, from birth to 240 days of age. Data from body weight and size were used for determining two formulae for calculating Body Mass Index (BMI). 

The first formula (BMI1) was extrapolated from human clinical studies:

Weight (kg)/ Length (m)2

The second formula (BMI2) introduced the trunk volume in the denominator:

$$\frac{\text{Weight (kg)}}{\pi \text{/3 x Length x [(TC/2}\pi \text{)}\text{2}\text{ + (AC/2}\pi \text{)}\text{2}\text{ + (TC/2}\pi \text{ x AC/2}\pi \text{)]}}$$

### Evaluation of changes in adiposity and plasma leptin concentration during juvenile development

**Figure 1:**
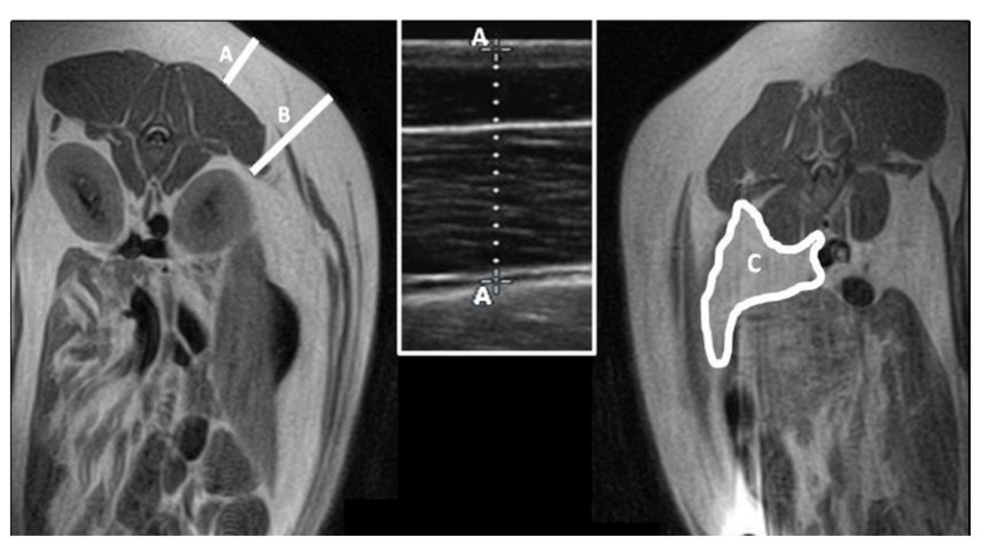
Imaging of subcutaneous and visceral fat depots. At the left hand, a Magnetic Resonance Imaging (MRI) of a transversal section of the swine body indicates the anatomical references for performing ultrasonographic (A) and MRI (B) evaluation of subcutaneous fatness. The ultrasonographic imaging of back-fat is represented in the inset at the centre of the image. At the right hand, the MRI image indicates the anatomical reference for evaluation of visceral fat depots.

Subcutaneous fat depth was evaluated monthly, from 60 to 240 days of age, by ultrasonography (Figure 1A); a SonoSite S-Series equipped with a 5-8MHz lineal array probe (SonoSite Inc., Bothell, WA) was used. The probe was placed against the skin, in a point at the right side of the animal located at 4cm from the midline and transversal to the head of the last rib as determined by palpation.

Visceral fatness was assessed at starting *ad libitum* access to the obesogenic diet (120 days) and at 180 days of age. Measurement of visceral fat content was performed by magnetic resonance imaging (MRI); subcutaneous adiposity was also determined when performing MRI scans, since MRI is highly adequate for visualization of both subcutaneous and visceral adipose tissue [12,13]. For MRI scanning, the gilts were anesthetized with isofluorane vapors (IsoFlo, Laboratorios Esteve, Barcelona, Spain), after sedation with xylazine (Rompun, Bayer Ag, Leverkusen, Germany) and ketamine (Imalgène 1000, Merial, Lyon, France), for minimizing stress and breathing movements during scans. MRI scans were carried out at the UCM Veterinary Teaching Hospital, by means of a Panorama 0.23T scanner with a body/spine XL coil (Philips Medical Systems, Best, Netherland). Animals were placed in lateral recumbence. Images were obtained in the transverse plane using a T1 weighted TSE (turbo spin-echo) sequence, from the thoracic inlet through the cranial margin of the ilium, and analyzed in a dedicated workstation using the ViewForum R6.3V1L3 software package (Philips Medical Systems, Best, Netherland). Measurements of subcutaneous fat depots were based on the maximum length obtained tracing a line perpendicular to the kidney at the level of the first lumbar vertebra. Values for visceral depots were calculated from axial areas obtained at the level of the third lumbar vertebra (Figure 1B and C).

Plasma concentrations of leptin were assessed concurrently with MRI scanning, when the pigs were 120 and 180 days-old. Blood samples were drawn by jugular venipuncture with 5 ml sterile heparin blood vacuum tubes (Vacutainer^TM^ Systems Europe). Immediately after recovery, the blood was centrifuged at 1500g for 15 min and the plasma was separated and stored into polypropylene vials at -20°C until assayed. Concentrations of leptin were determined in a single analysis using the Multi-species Leptin RIA kit (Demeditec Diagnostics GmbH, Kiel-Wellsee, Germany). The assay sensitivity was 1.0 ng ⁄ ml; the intra-assay variation coefficient was 3.1%.

### Evaluation of blood indexes of carbohydrates and lipids metabolism during juvenile development

Plasma indexes of carbohydrates and lipids metabolism were assessed, in blood samples obtained as previously described, monthly after starting *ad libitum* access to the fat diet (120 days). At 210 days of age, possible changes in beta cell function and insulin resistance were assessed by analyzing plasma insulin concentrations.

Glucose and fructosamine were measured by means of a clinical chemistry analyzer (Saturno 300 plus, Crony Instruments s.r.l., Rome, Italy) whilst insulin was determined by means of a Porcine Insulin ELISA kit (Mercodia AB, Uppsala, Sweden). The assay sensitivity was 0.26 UI ⁄l; the intra-assay variation coefficient was 3.5%. Possible changes in beta cell function and insulin resistance (IR) were assessed by the homeostasis model assessment (HOMA), using the equations HOMA-IR = (FINS x FPG)/22.5 to assess insulin resistance [14] and HOMA-β = (20 x FINS)/(FPG-3.5) to assess beta cell function [15]; FINS is fasting plasma insulin concentration in U/l and FPG is fasting plasma glucose concentration in mmol/l.

Triglycerides, total cholesterol, high-density lipoproteins cholesterol [HDL-c] and low-density lipoproteins cholesterol [LDL-c] were measured with the same analyzer (Saturno 300 plus). Plasma HDL-c ratio and LDL-c ratio were calculated by dividing HDL-c and LDL-c concentrations, respectively, by total cholesterol; plasma LDL-c/HDL-c ratio was obtained by dividing LDL-c levels by HDL-c concentrations. 

### Assessment of weight, corpulence, fatness and metabolic features at adulthood

The offspring reached target adult size and weight (around 110 kg body-weight for females and 120 kg body-weight for males), according to breed records, around 240 (OVERFED and UNDERFED groups) and 290 days of age (CONTROL and LATE-UNDERFED groups). At that time, assessment of phenotypic characteristics included weight, size, BMI1 and BMI2, subcutaneous and visceral adiposity, plasma levels of leptin and indexes of carbohydrates and lipids metabolism; which were determined as previously described.

### Analysis of fat composition at adulthood

At adulthood, females were kept in the experimental herd of INIA but all the males were euthanized and used for *ex vivo* evaluation of intramuscular fat composition. Samples of loin were obtained at the level of the last rib and vacuum-packaged in individual bags and stored at -20°C until analyzed.

Firstly, the fatty acids (FA) were extracted and quantified using the one-step procedure described by Sukhija and Palmquist [16] for lyophilized samples. Pentadecanoic acid (C15:0; Sigma, Alcobendas, Madrid, Spain) was used as the internal standard. Previously, methylated FA samples were identified according to Rey et al. [17] using a gas chromatograph (Model HP6890; Hewlett Packard Co., Avondale, PA, USA) and a 30m x 0.32mm x 0.25mm cross-linked polyethylene glycol capillary column (Hewlett Packard Innowax). A temperature program of 170°C to 245°C was used. The injector and detector were maintained at 250°C. The carrier gas (helium) flow rate was 3 ml/min. The lipids were obtained according to the method developed by Marmer and Maxwell [18]. Afterwards, fat extracts were methylated in the presence of sulphuric acid and analyzed as described above. From individual FA percentages, the saturated FA (SFA), monounsaturated FA (MUFA) and polyunsaturated FA (PUFA) proportions were calculated. SFA is the result of C10:0 + C12:0 + C14:0 + C15:0 + C16:0 + C17:0 + C18:0 + C20:0. MUFA is the result of C15:1 + C16:1n-9 + C17:1 + C18:1n-9 + C18:1n-7 + C20:1. PUFA is the result of C18:2n-6 + C18:3n-3 + C18:4n-3 + C20:3n-9 + C20:4n-6. Finally, the desaturation index (DI) is the ratio of MUFA to SFA.

### Statistical analyses

Changes on body weight and size, fat content and metabolic features over time were measured by Pearson correlation procedures. Effects of maternal diet and offspring sex on juvenile development were assessed by analysis of variance for repeated measures (split-plot ANOVA). Effects of maternal diet and offspring sex on adult phenotype were assessed by analysis of variance (two-way ANOVA), or by a Kruskall–Wallis test when a Levene’s test showed non-homogeneous variables, and a Duncan post hoc test was performed to contrast the differences among groups. Since all the variables changed over-time in a linear way, there were no differences between results of the correlation and ANOVA. Thus we will essentially report here ANOVA data Results were expressed as the mean ± SEM and statistical significance was accepted from P<0.05.

## Results

### Patterns of growth and fatness during juvenile development

**Figure 2:**
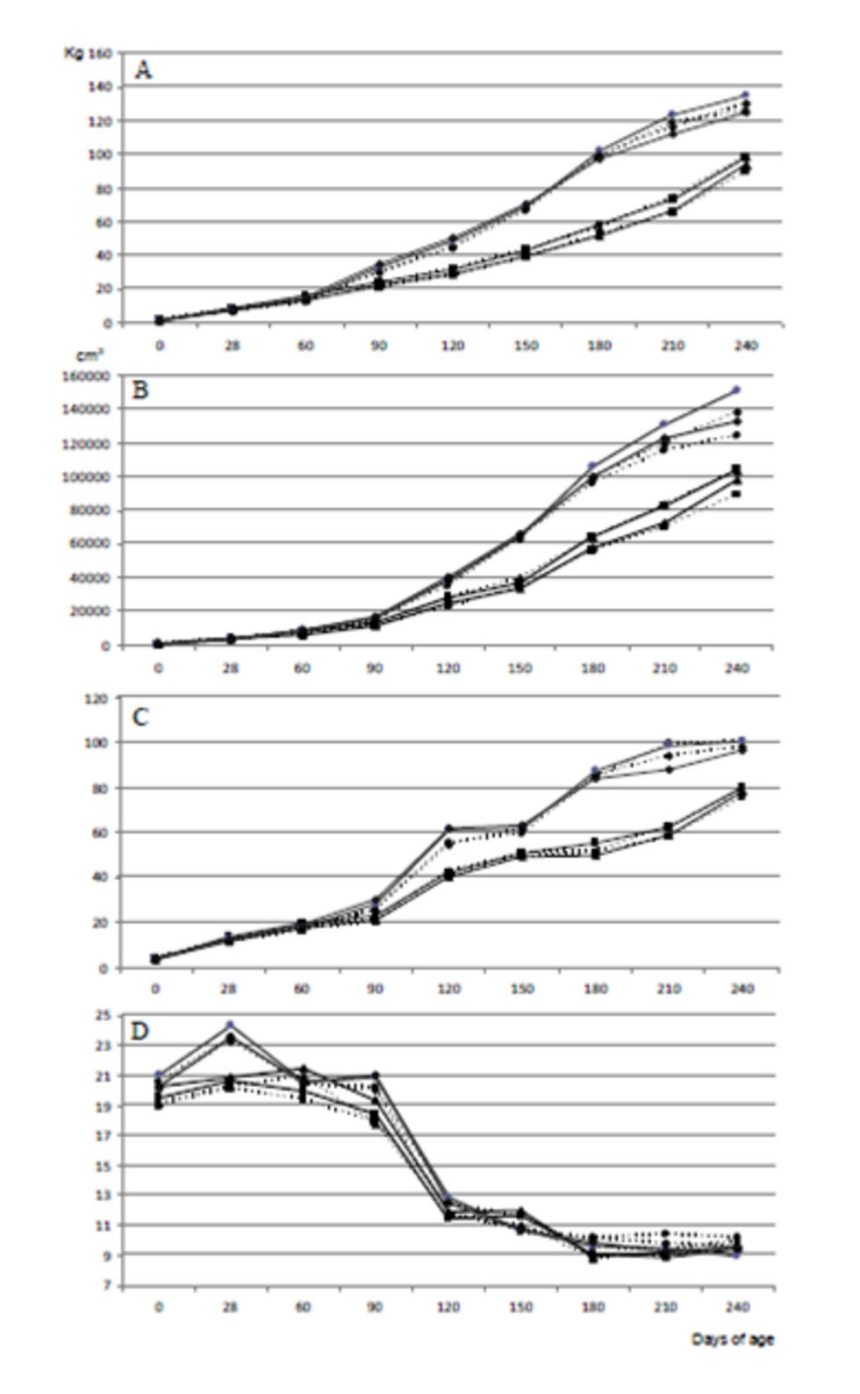
Effects of sex and maternal nutrition on offspring body growth. Changes over time in mean values for body weight (A), body volume (B) and Body Mass Indexes BMI1 (C) and BMI2 (D) in male (continuous line) and female (discontinuous line) Iberian piglets born from sows fed, during the entire pregnancy, with a diet fulfilling either 100% (CONTROL; black squares), or 160% (OVERFED; black circles) or 50% of daily maintenance requirements for gestation (UNDERFED; black diamonds). A fourth group (LATE-UNDERFED; black triangles) was born from females fed with 100% maintenance requirements until Day 35 of pregnancy, like the CONTROL group, but restricted to 50% of such amount from Day 36 onwards, like the UNDERFED group. Numerical data reported in the Table S1.

The maternal nutritional treatment affected body weight and size of the offspring, independently of sex, from the very first measurement at delivery (Day 0; Figure 2; numerical data reported in the Table S1). The piglets in the groups CONTROL, OVERFED and UNDERFED were similar among them (1.41 ± 0.01, 1.45 ± 0.01 and 1.46 ± 0.02 kg). On the other hand, all of them were around 10% larger and heavier than the piglets in the group LATE-UNDERFED (1.33 ± 0.01 kg; P<0.001). 

Afterwards, from 90 days of age and throughout the entire period of study, OVERFED and UNDERFED offspring were similar in weight between them and both groups grew faster and were significantly heavier than CONTROL pigs (P<0.0005). The pattern of postnatal growth was therefore influenced by maternal nutrition, but there was also found a significant effect of the sex of the offspring. In the CONTROL group, the males were always heavier than females during their juvenile development (P<0.005). Conversely, in the OVERFED and UNDERFED groups, offspring males and females were similar in weight during the whole study.

The effect of the sex of the piglet was even more evident in the LATE-UNDERFED group. At weaning, males were still lighter than those in the CONTROL group (P<0.05), but females reached a weight similar to their CONTROL counterparts. Afterwards, during juvenile development, LATE-UNDERFED females were heavier than CONTROL females (P<0.005) whilst, conversely, LATE-UNDERFED males were significantly lighter than CONTROL males (P<0.05). Again, differences in weight between LATE-UNDERFED males and females were not significant.

Changes over time in BMI1, trunk volume and BMI2 were also affected by maternal nutritional treatment (Figure 2; numerical data reported in the Table S1). Changes over time in BMI1 and trunk volume resembled the evolution in body weight. Values were similar in all the groups from birth to 90 days of age. From 120 days of age and throughout the entire period of study, the values were similar in OVERFED and UNDERFED offspring and significantly higher in both groups than in CONTROL and LATE-UNDERFED (P<0.0005 for both variables). On the other hand, the assessment of maternal effects on the BMI2 (the relationship between weight and trunk volume) indicated that, over time, the growth in volume was higher than the increase in weight in all the groups. Again, the values BMI2 were higher in OVERFED and UNDERFED than in CONTROL and LATE-UNDERFED offspring from 120 days of age onwards (P<0.05). However, the most remarkable differences were found in the evolution of BMI2 during lactation. At weaning, OVERFED and UNDERFED piglets showed a higher BMI2 than CONTROL and LATE-UNDERFED offspring (P<0.005), indicating a higher deposition of weight in relation with body development. 

There was, once more, a significant effect of offspring sex on these variables throughout the period of study. In the CONTROL group, males had higher BMI1 (P<0.005) and trunk volume (P<0.0005) than females; on the other hand, BMI2 was similar in both sexes. In the pigs from the OVERFED group, there was no difference in BMI1 between sexes but the trunk volume was higher in males (P<0.05). Hence, with similar body weights, BMI2 was higher in females (P<0.01). On the other hand, there were no sex differences in BMI1, nor in trunk volume, nor in BMI2 in the UNDERFED and LATE-UNDERFED groups, in spite of a trend (P=0.06) for a higher trunk volume in LATE-UNDERFED females when compared to males. 

**Figure 3:**
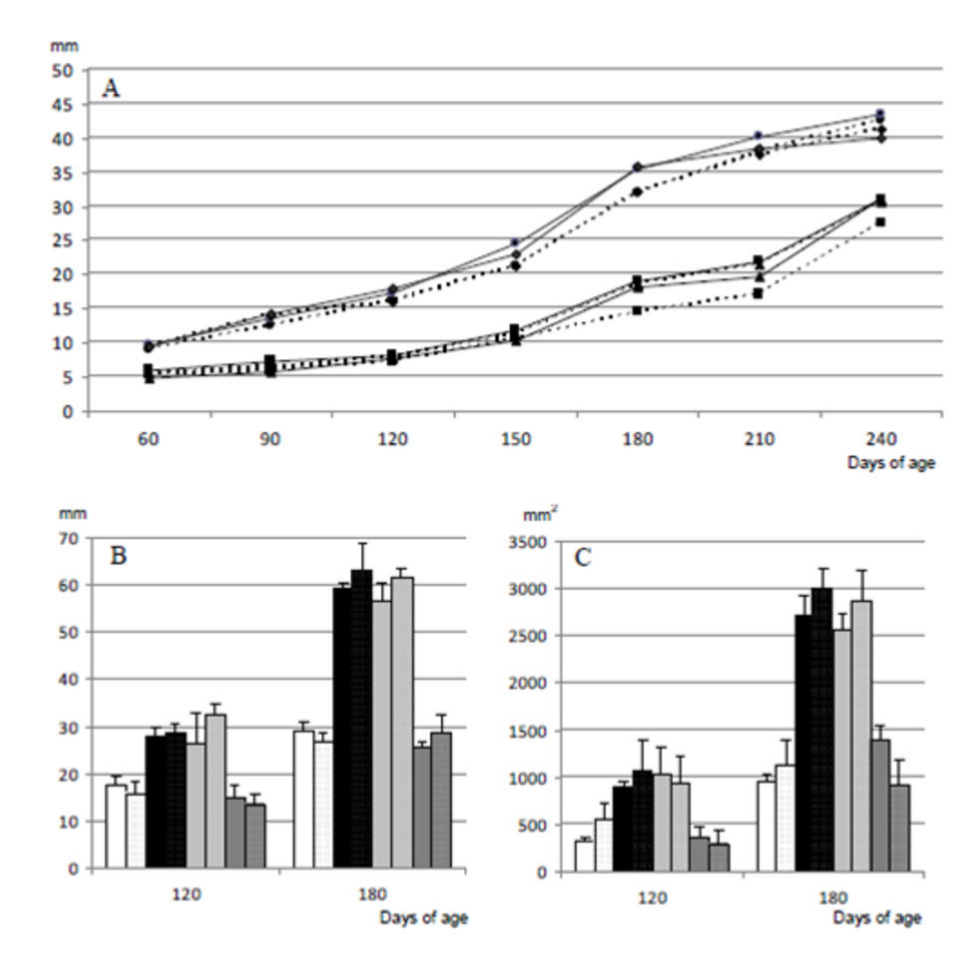
Effects of sex and maternal nutrition on offspring adiposity. Changes over time in mean values for subcutaneous back-fat depth, determined by ultrasonography (A) and MRI (B), and area of visceral fat depot at the level of the third lumbar vertebra (C) in male (continuous line and solid bars) and female (discontinuous line and dotted bars) Iberian piglets born from sows fed, during the entire pregnancy, with a diet fulfilling either 100% (CONTROL; black squares and white bars), or 160% (OVERFED; black circles and black bars) or 50% of daily maintenance requirements for gestation (UNDERFED; black diamonds and light-grey bars). A fourth group (LATE-UNDERFED; black triangles and dark-grey bars) was born from females fed with 100% maintenance requirements until Day 35 of pregnancy, like the CONTROL group, but restricted to 50% of such amount from Day 36 onwards, like the UNDERFED group. Numerical data reported in the Table S2.

In the same way, the values obtained by measuring the subcutaneous back-fat depth by ultrasonography, monthly from 60 to 240 days-old, were similar in OVERFED and UNDERFED offspring and significantly higher in these groups than in CONTROL and LATE-UNDERFED pigs (P<0.0005; Figure 3A; numerical data reported in the Table S2), without significant differences when comparing offspring from the same sex. Similar results were found when assessing both subcutaneous and visceral fat by MRI at 120 and 180 days-old (Figures 3B and 3C; numerical data reported in the Table S2).

On the other hand, changes in subcutaneous fat depth in the LATE-UNDERFED piglets were different between male and female littermates.. The LATE-UNDERFED and CONTROL females had similar values until 150 days of age; afterwards, subcutaneous fat was more abundant in LATE-UNDERFED females (P<0.01). Conversely, LATE-UNDERFED males showed lower back-fat depths than CONTROL counterparts until 180 days of age (P<0.05); afterwards, the values were similar in both groups. 

### Plasma leptin concentration during juvenile development

**Figure 4:**
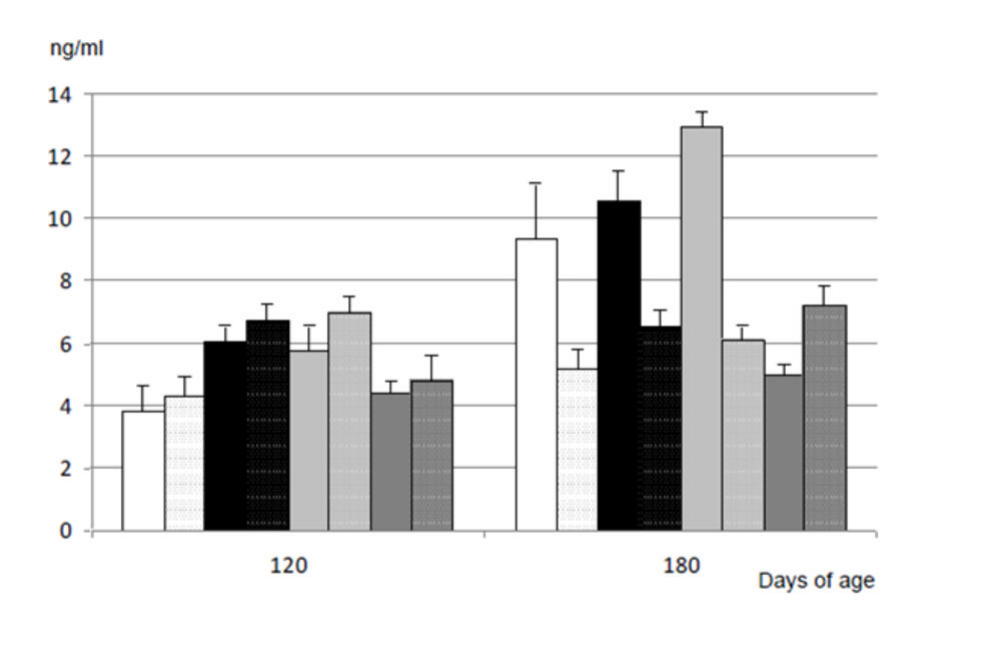
Effects of sex and maternal nutrition on plasma leptin concentration. Changes over time in mean values for plasma leptin concentrations (ng/ml) in male (solid bars) and female (dotted bars) Iberian piglets born from sows fed, during the entire pregnancy, with a diet fulfilling either 100% (CONTROL; white bars), or 160% (OVERFED; black bars) or 50% of daily maintenance requirements for gestation (UNDERFED; light-grey bars). A fourth group (LATE-UNDERFED; dark-grey bars) was born from females fed with 100% maintenance requirements until Day 35 of pregnancy, like the CONTROL group, but restricted to 50% of such amount from Day 36 onwards, like the UNDERFED group. Numerical data reported in the Table S3.

Plasma leptin concentrations increased with age and weight in all the groups (Figure 4; numerical data reported in the Table S3). At first assessment at 120 days-old, the values were similar in OVERFED and UNDERFED offspring and higher in both groups than in the CONTROL and LATE-UNDERFED pigs (P<0.01 for all the groups), which were similar between them. At 180 days-old, plasma leptin concentrations were still higher in the OVERFED and UNDERFED pigs than in the LATE-UNDERFED groups (P<0.01 for all the groups), but differences with CONTROL offspring did not reach statistical significance.

At 120 days of age, there was no significant effect of sex on plasma leptin concentrations in any of the groups, but at 180 days-old, CONTROL, OVERFED and UNDERFED females had lower leptin concentrations than their male littermates (P<0.05, P<0.005 and P<0.001, respectively). Conversely LATE-UNDERFED males had lower plasma leptin concentrations at 180 days of age than their sisters (P<0.005).

### Changes in plasma indexes of carbohydrates and lipids metabolism during juvenile development

Analysis of plasma indexes of carbohydrates metabolism showed differences among groups from the first sampling at 120 days of age (Table 1).Plasma glucose concentrations were similar in OVERFED and UNDERFED offspring and significantly higher in both groups than in CONTROL and LATE-UNDERFED piglets (P<0.0005 for all the groups) at 120 days of age. However, from 180 to 240 days of age, glycemia in OVERFED offspring was significantly higher than in the other three groups (P<0.05; P<0.005 at 240 days of age). There was no effect of sex in any of the groups.

**Table 1:** Effects of sex and maternal nutrition on offspring glucose metabolism.

		**CONTROL**		**OVERFED**		**UNDERFED**		**LATE-UNDERFED**		
	**Days of age**	**FEMALE**	**MALE**	**FEMALE**	**MALE**	**FEMALE**	**MALE**	**FEMALE**	**MALE**	**P**
**Glucose (mg/dl)**	**120**	77.3 ± 5.2	79.2 ± 3.2	116.4 ± 6.1	117.8 ± 4.7	104.2 ± 9.2	117.8 ± 6.6	77.9 ± 5.0	70.7 ± 4.0	0.000
	**150**	78.3 ± 3.8	72.2 ± 3.6	108.4 ± 3.3	105.4 ± 4.5	98.9 ± 5.6	103.4± 7.2	79.0 ± 4.3	80.0 ± 6.1	0.308
	**180**	92.3 ± 4.9	86.5 ± 6.4	102.1 ± 7.0	93.2 ± 3.6	94.8 ± 2.3	94.4 ± 6.7	88.4 ± 4.2	83.2 ± 3.9	0.068
	**210**	69.3 ± 8.0	73.1 ± 4.3	97.4 ± 20.4	75.4 ± 4.2	71.4 ± 3.7	79.2 ± 4.4	76.7 ± 5.7	73.1 ± 3.8	0.350
	**240**	76.7 ± 2.9	65.7 ± 2.6	80.7 ± 2.6	93.1 ± 6.6	71.6 ± 3.9	81.0 ± 5.2	83.4 ± 2.2	72.1 ± 4.9	0.004
**Insulin (U/l)**	**210**	1.3±0.1	1.3±0.1	1.8±0.2	1.8±0.1	1.4±0.2	1.5±0.1	1.4±0.1	1.6±0.2	0.026
**HOMA-β**	**210**	0.2±0.1	0.3±0.1	0.5±0.1	0.5±0.1	0.3±0.1	0.4±0.1	0.3±0.1	0.4±0.1	0.032
**HOMA-IR**	**210**	98.6±1.4	85.4±1.3	92.2±1.6	103.1±2.1	83.4±1.4	95.9±1.6	83.1±1.2	94.0±1.2	0.169
**Fructosamine (mg/dl)**	**120**	211.4 ± 8.8	216.6 ± 7.7	210.7 ± 9.3	224.0± 7.0	221.1 ± 8.6	200.2 ± 12.6	200.9 ± 10.9	201.3 ± 6.6	0.240
	**150**	202.9 ± 8.5	214.4 ± 4.7	223.4 ± 9.3	243.5± 5.6	241.6 ± 6.5	246.5 ± 10.2	209.9 ± 8.4	220.9 ± 5.9	0.324
	**180**	204.1 ± 4.1	210.9 ± 5.5	248.0 ± 8.7	250.0 ± 12.9	260.1 ± 3.3	264.2 ± 14.3	204.7 ± 13.2	211.7 ± 4.5	0.000
	**210**	210.3 ± 5.5	215.7 ± 4.5	229.0 ± 21.9	254.9 ± 8.2	262.6 ± 5.5	240.7 ± 14.0	232.3 ± 14.7	201.4 ± 9.0	0.002
	**240**	220.3 ± 12.1	224.7 ± 11.8	234.8 ± 5.5	234.9 ± 9.1	236.1 ± 2.6	222.2 ± 9.7	226.9 ± 10.3	218.5 ± 7.3	0.038

Changes over time in mean values (± S.E.M.) for glucose and fructosamine, and values for insulin and HOMA indexes, in male and female pigs born from sows fed, during the entire pregnancy, with a diet fulfilling 100% (CONTROL), 160% (OVERFED) or 50% of daily maintenance requirements for gestation (UNDERFED). A fourth group (LATE-UNDERFED) was born from females restricted to 50% of such amount from Day 36 onwards.

Assessment of plasma insulin concentrations also showed different values among groups (Table 1). Overall, OVERFED piglets showed higher values than UNDERFED and LATE-UNDERFED offspring (P<0.05 with both groups). The three groups showed higher values than CONTROL pigs (P<0.05 for UNDERFED and LATE-UNDERFED; P<0.01 for OVERFED). Assessment of HOMA-IR index showed higher values in OVERFED than in the CONTROL group (P<0.05); values in the other groups were not significantly different from CONTROL piglets. On the other hand, HOMA-β indexes were similar among treatments. In the OVERFED group, the male piglets showed higher values of plasma insulin and, thus, a higher HOMA-IR index than their female counterparts (P<0.05 for both variables); there was no effect of sex in the other groups.

Analysis of plasma fructosamine concentrations showed no differences among groups at 120 days of age (Table 1). However, from 180 to 240 days of age, fructosamine values were similar in OVERFED and UNDERFED piglets and significantly higher in both groups than in CONTROL and LATE-UNDERFED offspring (P<0.0005 at 180 days of age; P<0.05 at 210 and 240 days of age). There was no effect of sex in any of the groups.

Screening of parameters related to lipid metabolism also showed significant effects of maternal dietary treatments, with similar differences in plasma concentrations of triglycerides, cholesterol and LDL-c and HDL-c among groups (Table 2). From 120 to 210 days of age, OVERFED piglets showed higher plasma concentrations of triglycerides and cholesterol than UNDERFED (P<0.05) and LATE-UNDERFED and CONTROL offspring (P<0.01 with both groups). At 240 days of age, the same differences were found in cholesterol levels, but there were no significant differences in triglycerides concentrations. These higher levels of cholesterol in the OVERFED piglets were caused by higher concentrations of both HDL-c and LDL-c at 120 and 180 days and by higher concentrations of only LDL-c from 210 days of age. However, no differences in the LDL-c/HDL-c ratio were observed.

**Table 2:** Effects of sex and maternal nutrition on offspring lipids metabolism.

		**CONTROL**		**OVERFED**		**UNDERFED**		**LATE-UNDERFED**		
	**Days of age**	**FEMALE**	**MALE**	**FEMALE**	**MALE**	**FEMALE**	**MALE**	**FEMALE**	**MALE**	**P**
**Triglycerides (mg/dl)**	**120**	46.7 ± 4.5	55.6 ± 7.4	81.7 ± 13.7^a^	116.9 ± 10.9^b^	61.3 ± 10.2^a^	94.2 ± 9.8^b^	46.6 ± 6.1	48.2 ± 6.4	0.000
	**150**	38.4 ± 5.5	40.0 ± 2.9	76.6 ± 12.4	101.9 ± 8.0	60.2 ± 6.4	62.3 ±8.9	44.0 ± 7.5	39.8 ± 5.1	0.066
	**180**	46.9 ± 6.7	57.4 ± 7.6	66.4 ± 8.6	74.2 ± 4.2	58.4 ± 4.2	42.8 ± 10.9	50.9 ± 8.8	58.2 ± 4.8	0.033
	**210**	44.3 ± 8.9	43.5 ± 3.8	61.0 ± 11.7	80.0 ± 13.6	51.2 ± 6.3	63.2 ± 9.4	39.1 ± 4.2	36.8 ± 3.9	0.001
	**240**	62.9 ± 7.3^a^	96.8 ± 16.6^b^	64.4 ± 7.9	83.7 ± 12.4	59.7 ± 10.2	63.8 ± 11.5	57.4 ± 14.4	77.6 ± 16.9	0.474
**Total cholesterol (mg/dl)**	**120**	89.1 ± 5.3	101.8 ± 5.3^b^	136.2 ± 12.9	140.7 ± 5.7	119.1 ± 10.1	112.1 ± 7.7	80.6 ± 4.8	93.0 ± 3.8	0.000
	**150**	96.8 ± 3.9 ^a^	102.4 ± 4.6^b^	123.4 ± 9.3	139.1 ± 11.0	114.6 ± 7.2	118.4 ± 8.8.	98.9 ± 4.6	98.5 ± 6.2	0.078
	**180**	91.7 ± 4.5^a^	112.8 ± 5.5^b^	117.1 ± 7.5	131.6 ± 6.8	109.6 ± 5.3	126.6 ± 6.0	106.5 ± 5.8	108.0 ± 3.7	0.005
	**210**	88.7 ± 7.9^a^	106.9 ± 6.8^b^	124.3 ± 8.1	124.7 ± 7.9	98.3 ± 5.5	109.8 ± 2.9	91.9 ± 8.9	100.0 ± 5.0	0.000
	**240**	97.5 ± 4.2^a^	105.1 ± 5.2^b^	100.6 ± 6.1	113.1 ± 7.8	85.1 ± 5.4	94.3 ± 6.9	98.7 ± 6.3	99.5 ± 6.4	0.043
**HDL-cholesterol (mg/dl)**	**120**	24.0 ± 4.8	33.5 ± 1.7	41.0 ± 5.2	44.7± 3.9	31.7 ± 6.3	35.7 ± 3.2	22.1 ± 4.1	24.4 ± 2.9	.000
	**150**	31.2 ± 1.8	33.3 ± 1.7	39.8 ± 4.9	45.2 ± 3.1	36.5 ± 5.3	37.4 ± 4.2	33.91 ± 5.0	33.4 ± 2.9	.692
	**180**	28.0 ± 1.8	28.5 ± 3.2	34.5 ± 4.9	45.6 ± 2.4	39.6 ± 4.6	39.1 ± 5.0	28.9 ± 4.1	27.0 ± 2.5	.001
	**210**	30.6 ± 5.2	34.8 ± 2.9	40.1 ± 4.3	34.0 ± 6.4	30.4 ± 5.0	33.3 ± 3.0	28.1 ± 8.2	27.9 ± 3.6	.302
	**240**	30.8 ± 4.2	27.6 ± 4.7	22.3 ± 4.6	33.1 ± 2.8	24.4 ± 3.8	30.2 ± 5.4	32.2 ± 7.2	23.9 ± 3.4	.970
**LDL-cholesterol (mg/dl)**	**120**	55.8 ± 3.7	57.2 ± 4.4	78.8 ± 9.7	72.6 ± 5.3	76.1 ± 8.4	57.6 ± 5.5	49.2 ± 3.3	59.0 ± 2.6	0.002
	**150**	57.9 ± 3.7	61.1 ± 4.0	72.1 ± 7.8	76.5 ± 6.7	62.4 ± 6.7	64.5 ± 7.8	56.2 ± 4.1	57.2 ± 3.4	0.431
	**180**	54.4 ± 3.8	72.7 ± 6.4	64.9 ± 5.7	78.7 ± 10.0	58.3 ± 5.4	78.9 ± 10.2	67.4 ± 6.2	69.3 ± 4.2	0.757
	**210**	50.2 ± 6.6	63.6 ± 6.5	72.1 ± 6.6	74.6 ± 4.6	57.6 ± 5.6	63.9 ± 1.9	56.0 ± 4.8	64.7 ± 4.4	0.031
	**240**	54.2 ± 2.5	58.1 ± 4.9	65.5 ± 3.7	63.3 ± 8.5	48.8 ± 3.0	51.4 ± 5.2	55.0 ± 5.2	60.1 ± 4.3	0.047

Changes over time in mean values (± S.E.M.) for triglycerides, total cholesterol, HDL-cholesterol and LDL-cholesterol in male and female pigs born from sows fed, during the entire pregnancy, with a diet fulfilling 100% (CONTROL), 160% (OVERFED) or 50% of daily maintenance requirements for gestation (UNDERFED). A fourth group (LATE-UNDERFED) was born from females restricted to 50% of such amount from Day 36 onwards. Different superscripts within the same nutritional group indicate significant differences between sexes (a≠b: P<0.05).

In the CONTROL group, plasma cholesterol was always higher in males than in females (P<0.05); such difference was not observed in the other groups. Moreover, there was no effect of sex on HDL-c and LDL-c. With regards to triglycerides, OVERFED and UNDERFED males showed higher values than females at 120 days of age (P<0.05); no effect of sex was detected in the other two groups at this age. Afterwards, there was no significant effect of sex in any of the groups excepting higher values in CONTROL males than females at 240 days (P<0.05).

### Weight, body-size, adiposity, plasma leptin concentration and fat composition at adulthood

Table 3 summarizes phenotypic values found when pigs reached adult size (at 240 days of age in OVERFED and UNDERFED pigs and at 290 days of age in CONTROL and LATE-UNDERFED pigs). 

**Table 3:** Effects of sex and maternal nutrition on body size and fatness at adulthood.

	**CONTROL**		**OVERFED**		**UNDERFED**		**LATE-UNDERFED**		
	**FEMALE**	**MALE**	**FEMALE**	**MALE**	**FEMALE**	**MALE**	**FEMALE**	**MALE**	**P**
**Body weight (kg)**	110.1 ± 2.2^e^	121.4 ± 2.2^f^	125.4 ± 4.2^e^	134.3 ± 3.3^f^	129.9 ± 3.1	124.8 ± 2.4	116.9 ± 5.0	113.7 ± 2.2	0.000
**Trunk volume (m^3^)**	117.5 ± 1.4^c^	142.3 ± 9.0^d^	123.7 ± 5.4^c^	150.6 ± 6.8^d^	137.8 ± 4.3	132.5 ± 3.4	131.0 ± 5.3	127.6 ± 3.6	0.592
**BMI1**	85.5 ± 1.7^e^	91.5 ± 1.1^f^	100.3 ± 3.1^e^	100.4 ± 1.8^f^	98.0 ± 2.8	96.3 ± 2.7	87.8 ± 3.2	87.3 ± 1.3	0.000
**BMI2**	9.4 ± 0.15	8.7 ± 0.3	10.2 ± 0.3^a^	8.9 ± 0.2^b^	9.5 ± 0.3	9.6 ± 0.2	8.9 ± 0.2	8.9 ± 0.1	0.028
**Subcutaneous fatness (US; mm)**	38.4 ± 4.1	45.3 ± 1.8	42.5 ± 1.3	43.6 ± 1.9	41.3 ± 0.2	40.0 ± 1.4	45.6 ± 1.2	49.1 ± 1.6	0.348
**Subcutaneous fatness (MRI; mm)**	70.9±1.8	76.6 ± 3.1	70.1 ± 3.3	78.3 ± 6.9	70.8 ± 4.7	73.3 ± 3.8	65.9 ± 4.7	70.9 ± 3.0	0.540
**Visceral fatness (MRI; cm^2^)**	29.5 ± 1.3	24.2 ± 5.6	37.4 ± 3.2	38.5 ± 5.0	37.8 ± 0.8^a^	29.5 ± 2.6^b^	38.0 ± 2.8^a^	24.6 ± 3.2^b^	0.015
**Intramuscular fatness (%)**	-	19.4 ± 1.7	-	25.1 ± 2.4	-	24.2 ± 0.9	-	15.4 ± 1.1	0.001

Morphological features (mean ± S.E.M.) of male and female pigs born from sows fed, during the entire pregnancy, with a diet fulfilling 100% (CONTROL), 160% (OVERFED) or 50% of daily maintenance requirements for gestation (UNDERFED). A fourth group (LATE-UNDERFED) was born from females restricted to 50% of such amount from Day 36 onwards. Different superscripts within the same nutritional group indicate significant differences between sexes (a≠b: P<0.05; c≠d: P<0.01; e≠f: P<0.005).

The maternal nutritional treatment and the offspring sex continued modulating body weight and size at adulthood. OVERFED and UNDERFED pigs were, overall, heavier and more corpulent than CONTROL and LATE-UNDERFED pigs (P<0.001 for all the groups). In the CONTROL and OVERFED groups, males had higher weight and BMI1 than females (P<0.005); conversely, there was no difference between males and females in the other two groups. 

The values for subcutaneous fat depth were similar in all the groups, as detailed in Table 3. However, the values for visceral fat depots continued being higher in OVERFED and UNDERFED than in CONTROL pigs (P<0.05). The values in the UNDERFED and LATE-UNDERFED pigs were determined by the sex. Males were similar to CONTROL counterparts, but females showed similar values to OVERFED gilts, so higher than in CONTROL females (P<0.05). The percentage of intramuscular fat, as evaluated by histology in the males, was also higher (P<0.001) in OVERFED and UNDERFED than in CONTROL and LATE-UNDERFED pigs.

Plasma leptin concentrations were again similar when comparing OVERFED and UNDERFED offspring; in the same way, values were also similar when comparing CONTROL and LATE-UNDERFED pigs (Table 4). Such values were significantly higher in both OVERFED and UNDERFED than in CONTROL and LATE-UNDERFED pigs (P<0.05 for all the groups). There were no significant effects of sex on plasma leptin concentrations of CONTROL and LATE-UNDERFED piglets but UNDERFED females had higher values than male counterparts (P<0.05). On the other hand, leptin concentration was higher in OVERFED males than in their sisters (P<0.05).

**Table 4:** Effects of sex and maternal nutrition on plasma leptin concentration at adulthood.

	**CONTROL**		**OVERFED**		**UNDERFED**		**LATE-UNDERFED**		
	**FEMALE**	**MALE**	**FEMALE**	**MALE**	**FEMALE**	**MALE**	**FEMALE**	**MALE**	**P**
**Leptin (ng/ml)**	11.9±4.3	12.5±2.5	14.7±0.6	18.2±3.1	20.6±3.2	11.0±0.9	10.9±2.8	13.9±2.4	0.019

Mean values (± S.E.M.) for plasma leptin concentration in male and female pigs born from sows fed, during the entire pregnancy, with a diet fulfilling 100% (CONTROL), 160% (OVERFED) or 50% of daily maintenance requirements for gestation (UNDERFED). A fourth group (LATE-UNDERFED) was born from females restricted to 50% of such amount from Day 36 onwards.

The maternal nutritional treatments also led to differences in fat composition (Table 5). The proportion of saturated fatty acids (SFA) was higher (P<0.001) in CONTROL and LATE-UNDERFED than in OVERFED and UNDERFED pigs due to a higher content in C14:0, C17:0, C18:0, C20:0 (P<0.01 for all) and C16:0 (P<0.05). On the other hand, the proportion of monounsaturated fatty acids (MUFA) was higher (P<0.001) in OVERFED and UNDERFED pigs than in the other two groups due to a higher content in C16:1, C18:1n-7 and C18:1n-9 (P<0.01 for all). There were no significant differences in the proportion of polyunsaturated fatty acids (PUFA) among treatments, although these values tended to be lower in OVERFED pigs and higher in the LATE-UNDERFED group. Finally, the FA desaturation index was higher in the three groups of pigs that were born from nutritionally treated sows than in the CONTROL pigs (P<0.05 for OVERFED and LATE-UNDERFED; P<0.01 for UNDERFED); without differences among these three groups.

**Table 5:** Effects of sex and maternal nutrition on fat composition at adulthood.

	**CONTROL**	**OVERFED**	**UNDERFED**	**LATE-UNDERFED**	**P**
**C10_0**	0.07±0.00	0.11±0.01	0.12±0.01	0.08±0.00	0.000
**C12_0**	0.07±0.00	0.08±0.01	0.08±0.00	0.07±0.00	0.682
**C14_0**	1.53±0.02	1.46±0.04	1.31±0.02	1.50±0.04	0.002
**C14_1**	0.15±0.01	0.14±0.01	0.15±0.01	0.16±0.01	0.453
**C15_1**	0.50±0.07	0.61±0.05	0.73±0.10	0.70±0.07	0.082
**C16_0**	26.13±0.31	25.10±0.28	24.41±0.28	25.60±0.29	0.007
**C16_1**	3.74±0.08	4.36±0.10	3.88±0.07	3.78±0.09	0.000
**C17_0**	0.23±0.01	0.18±0.01	0.21±0.02	0.22±0.01	0.000
**C17_1**	0.25±0.01	0.19±0.01	0.22±0.01	0.24±0.01	0.000
**C18_0**	13.57±0.26	10.91±0.21	11.03±0.07	13.18±0.24	0.000
**C18_1n-9**	42.83±0.55	46.58±0.50	47.54±0.39	42.91±0.47	0.000
**C18_1n-7**	3.44±0.08	3.79±0.10	3.58±0.13	3.35±0.04	0.000
**C18_2n-6**	4.99±0.31	4.38±0.20	4.72±0.35	5.61±0.37	0.049
**C18_3**	0.17±0.01	0.12±0.01	0.10±0.03	0.16±0.02	0.008
**C20_0**	0.19±0.00	0.16±0.00	0.17±0.01	0.18±0.01	0.000
**C20_1**	0.82±0.01	0.83±0.02	0.86±0.01	0.76±0.02	0.007
**C20_2**	0.21±0.01	0.24±0.01	0.26±0.01	0.22±0.01	0.053
**C20_3**	0.98±0.10	0.93±0.08	1.08±0.16	1.24±0.14	0.075
**C20_5**	0.29±0.04	0.28±0.04	0.27±0.04	0.27±0.04	0.327
**SFA**	41.80±0.56	37.96±0.41	37.53±0.61	40.79±0.50	0.000
**MUFA**	51.73±0.55	56.37±0.49	56.81±0.33	51.90±0.48	0.000
**PUFA**	6.47±0.40	5.66±0.28	6.16±0.50	7.32±0.48	0.048
**FADI**	66.17±0.91	68.75±0.55	70.31±0.83	68.26±0.97	0.032

Mean values (± S.E.M.) for individual fatty acids (FA), proportions of saturated FA (SFA), monounsaturated FA (MUFA) and polyunsaturated FA (PUFA) and desaturation index (FADI) in male pigs born from sows fed, during the entire pregnancy, with a diet fulfilling 100% (CONTROL), 160% (OVERFED) or 50% of daily maintenance requirements for gestation (UNDERFED). A fourth group (LATE-UNDERFED) was born from females restricted to 50% of such amount from Day 36 onwards.

### Indexes of carbohydrates and lipids metabolism at adulthood

Assessment of plasma indexes of carbohydrates metabolism showed, overall, higher values of glucose in OVERFED pigs (P<0.005) than in the UNDERFED and LATE-UNDERFED groups (Table 6). There were no significant effects of offspring sex in any of the groups. Plasma fructosamine concentrations were similar in OVERFED and UNDERFED piglets and significantly higher in both groups than in CONTROL and LATE-UNDERFED offspring (P<0.0005). 

**Table 6:** Effects of sex and maternal nutrition on glucose and lipid metabolism at adulthood.

	**CONTROL**		**OVERFED**		**UNDERFED**		**LATE-UNDERFED**		
	**FEMALE**	**MALE**	**FEMALE**	**MALE**	**FEMALE**	**MALE**	**FEMALE**	**MALE**	**P**
**Glucose (mg/dl)**	88.6 ± 4.8	96.3 ± 5.3	80.7 ± 2.6	93.1 ± 6.6	71.6 ± 3.9	81.0 ± 5.2	92.7 ± 6.2	89.9 ± 3.1	0.004
**Fructosamine (mg/dl)**	201.9 ± 12.7	203.2 ± 12.0	234.8 ± 5.5	234.9 ± 9.1	236.1 ±2.6	222.2 ± 9.6	189.9 ± 5.2^a^	218.3 ± 6.1^b^	0.000
**Triglycerides (mg/dl)**	48.0 ± 9.1	87.1 ± 11.8	64.4 ± 7.9	83.7 ± 12.4	59.7 ± 10.2	63.8 ± 11.5	58.7 ± 12.5	44.1 ± 7.4	0.123
**Total cholesterol (mg/dl)**	98.5 ± 6.6^a^	121.8 ± 9.5^b^	100.6 ± 6.1 ^a^	113.1 ± 7.8^b^	85.1 ± 5.4^a^	94.3 ± 6.9^b^	97.7 ± 8.4^a^	122.4 ± 6.0^b^	0.016
**HDL-c (mg/dl)**	34.8 ± 4.3^a^	46.2 ± 7.8^b^	22.3 ± 4.6^a^	33.1 ± 2.7^b^	24.9±3.8^a^	30.2 ± 5.4^b^	24.5 ± 3.0^a^	48.9 ± 3.9^b^	0.013
**LDL-c (mg/dl)**	54.1 ± 3.7	58,2 ± 4.1	65.5 ± 3.8	63.3 ± 8.5	48.9 ± 3.0	51.4 ± 5.2	61.5 ± 7.6	61.6 ± 5.0	0.044

Mean values (± S.E.M.) for glucose, fructosamine, triglycerides, total cholesterol, HDL-cholesterol and LDL-cholesterol in male and female pigs born from sows fed, during the entire pregnancy, with a diet fulfilling 100% (CONTROL), 160% (OVERFED) or 50% of daily maintenance requirements for gestation (UNDERFED). A fourth group (LATE-UNDERFED) was born from females restricted to 50% of such amount from Day 36 onwards. Different superscripts within the same nutritional group indicate significant differences between sexes (a≠b: P<0.05).

Analysis of parameters related to lipid metabolism (Table 6) showed a lack of differences among groups in plasma concentrations of triglycerides. On the other hand, plasma concentrations of total cholesterol were affected by maternal nutritional treatment. The OVERFED pigs showed lower concentrations of HDL-c and higher concentrations of LDL-c than all the other groups (P<0.05). Thus, OVERFED pigs had significantly a higher LDL-c/HDL-c ratio than CONTROL and LATE-UNDERFED offspring (P<0.05). On the other hand, differences with UNDERFED were not significant, since this group also had a higher LDL-c/HDL-c ratio than CONTROL and LATE-UNDERFED offspring (P<0.05).

Concentrations of fructosamine were higher in LATE-UNDERFED males than females (P<0.05). In all the groups, total cholesterol and HDL-c were also higher in males (P<0.05).

## Discussion

The results of the present study indicate that the intrauterine exposition of conceptuses to maternal malnutrition is associated with significant changes in their early postnatal and juvenile development, metabolism and, hence, in their subsequent adult phenotype. These changes are modulated mainly by type (deficiency or excess) and timing (entire pregnancy or only the last two thirds of gestation) of maternal malnutrition, but there was also found a strong modulatory effect of the offspring sex.

The differences in growth patterns and adult phenotype related to maternal nutrition and offspring sex found in this trial are similar to previous studies in human beings and laboratory animals [19]. These results support the validity of the Iberian pig as a robust, amenable and reliable translational model for studies on nutrition-associated diseases. In the present study, the effects of prenatal programming were found after feeding the Iberian sows with diets more moderate than reported in other swine models [20-22], like we have previously found in dietary treatments for inducing obesity and associated disorders at juvenile and adult stages [4,5]. These unique features can be related to the background of exposure to harsh environments and food scarcity for generations of the Iberian pig. Thus, the results found in the present experiment must be considered of importance for increasing the knowledge on the factors involved in the propensity of people living in developing countries for undergoing obesity and associated diseases.

A possible weakness of the current study, arising from an experimental point of view, is the lack of a fifth group assessing features of offspring exposed to maternal overnutrition during the last two thirds of pregnancy. However, from a translational point of view, such group is useless; diets may be restrained in pregnant females after diagnosis for avoiding excesses in weight gain throughout gestation, although this is a very controversial issue, but they are not usually exposed to increases in food intake over pregnancy necessities after confirmation of pregnancy.

### Effects of maternal nutrition on growth and fatness of the offspring

The results of the current study indicate, firstly, that the newborns from sows exposed to either under- or overnutrition during the entire gestation have a similar body weight and size than control neonates. Such findings, similar to previous data from epidemiological studies in humans [23-28] and experimental approaches in animals [29-32], gives new evidences favoring the concept of the adaptive response of embryos to maternal malnutrition [19]. Moreover, these results support the hypothesis of Watkins and co-workers [33] that, in case of consistency between pre- and post-implantation periods, adaptive responses induced in the embryos by maternal nutrition led to normal fetal growth. On the other hand, inconsistency in food availability between both periods led to alterations in the growth of the conceptuses. In agreement with this concept, in the current study, newborns from sows exposed to undernutrition during only the last two thirds of pregnancy have a significantly lower body weight and size than control counterparts; results that are also in agreement with previous studies in humans and animal models and, specifically, in the pig [34-39].

Afterwards, reinforcing the concept of DOHaD [10,11], the piglets exposed to both under- and overnutrition during the entire pregnancy have increased growth patterns during early postnatal and juvenile development, developing higher body weights, sizes and fatness than individuals from pregnancies underfed at the last two thirds and even than the control offspring. These differences were found from 90 days of age; i.e.: prior to exposure to postnatal obesogenic diet (120 days of age). Thus, it is not necessary a postnatal excess of nutrients for triggering the disruptive effects of prenatal programming, at least in the Iberian pig. However, the exposure of these individuals to obesogenic diets is also related to higher rates of fat accumulation and, hence, favours the onset of obesity. Moreover, it is noteworthy the difference among groups in the evolution of the ratio between body weight and volume (BMI2) during lactation: an outstanding result of the present study. Although there are no significant differences in body weight and size among the piglets exposed to either under- or overnutrition during the entire pregnancy and the control individuals, BMI2 at weaning indicates a higher weight deposition in relationship to body development in the piglets exposed to maternal malnutrition during the entire pregnancy. Such finding strengths the role of prenatal programming on infant development and encourages the necessity of further research in this area.

### Effects of offspring sex on postnatal growth and fatness

All the effects of maternal malnutrition reported in the current study are modulated by the sex of the offspring. The comparison of control males and females littermates indicates that males are always heavier and larger than females. However, conversely, females from under- and overfed pregnancies have enhanced growth patterns and, hence, body weight, corpulence and adiposity, which are similar to those of male littermates. The effect of the sex of the offspring on postnatal growth is even more evident in the individuals from pregnancies underfed at the last two thirds. The males from late underfed sows continue having lower weight size and adiposity than control males during the entire juvenile development. Conversely, the females undergo enhanced postnatal growth and reach higher weight than control females at a so early moment as at weaning time and continue gaining more weight than their brothers during the entire juvenile period. These data indicate sex-specific differences in postnatal growth in the Iberian breed that have not been previously reported in lean swine [40,41]. Overall, all the features described above indicate a predisposition for a better postnatal development of the Iberian females, which are more critical for the survival of the species with an enhanced body development in females from underfed sows occurring even prior to exposure to postnatal obesogenic diet. However, in the case of postnatal food abundance, the consequences of prenatal programming are even more negative. In our study, the exposure of females from underfed pregnancies to obesogenic diets is related to an increased fat deposition; hence, at the end of the growth period, these females have significantly higher fat depots than control females.

The results of the present study, taken as a whole, give new evidences to the theory of DOHaD. Moreover, the serial screening of changes in the metabolic features of the offspring allowed the finding of non-previously described effects of maternal nutrition. On the other hand, the influence of the offspring sex on metabolic features was weaker, although indexes were always augmented in males. The effects of maternal nutrition found in the present trial were displayed even with controlled postnatal food intake, but enhanced after exposure to obesogenic diets during their juvenile development as described in Iberian females that were not exposed to prenatal programming [5]. 

### Effects of maternal nutrition on offspring predisposition to metabolic syndrome

Serial assessment of adiposity, performed by ultrasonography, magnetic resonance imaging and, finally, post-mortem, indicates that offspring from sows over- and underfed during the entire pregnancy have increased fat depots both at subcutaneous and visceral levels during juvenile development. At adulthood, differences in subcutaneous adiposity are vanished, probably as a consequence of the obesogenic diet. However, pigs exposed to prenatal malnutrition have still a significant higher amount of fat at visceral and intramuscular location. This feature indicates that these individuals have developed *central obesity*; the first symptom of the metabolic syndrome. 

The metabolic syndrome is the main risk factor for developing type-2 diabetes and cardiovascular diseases [42,43]. The syndrome is characterized by the presence of at least three of five symptoms: central obesity, impaired glucose regulation, insulin resistance, dyslipidemia (increased triglyceridemia and low plasma HDL-c), and hypertension [44–47]. We did not assessed cardiovascular features in the present study, but evaluation of the metabolic status showed significant effects of maternal malnutrition on glucose and lipids metabolism. 

First, the individuals from the three groups exposed to maternal malnutrition during pregnancy (either by over- or undernutrition during the entire pregnancy or its last two thirds) are affected by *impairments of glucose regulation*, with increased *insulin secretion* in order to maintain adequate plasma glucose concentrations, like it is found in human beings [48]. However, plasma fructosamine concentrations remained higher in offspring exposed to maternal malnutrition during the entire pregnancy. Possibly, alterations of glucose regulation in these piglets are related to increases in fat-content, since it is well-known, in human young individuals, that adiposity increases insulin resistance [48,49]. This relationship between adiposity and insulin resistance found in human adolescents seems to be related to increased plasma leptin concentrations; in fact, although adipokines are involved in the prodrome of diabetes [50], leptin is the only adipokine reported to correlate with insulin resistance in children to the date [50]. Moreover, leptin relationships with metabolic alterations and type-2 diabetes are strongly affected by the genetic type, the breed, both at infant and adult stages [51,52]. 

The three groups exposed to maternal malnutrition during pregnancy showed impairments of glucose regulation and increased insulin secretion, but we should note that these alterations get worsened in the offspring from overfed pregnancies. These piglets have a clearly established *insulin resistance* as indicated by the HOMA-IR index, although impairments of β-cell function are still not established at such a young age. However, consequences may be found in a later age since previous results in adult Iberian sows evidenced the propensity of the breed to develop type-2 diabetes [4]. In fact, chronic consumption of obesogenic diets induces a progressive deterioration in secretion of insulin by β-cells [53] which may give way to type-2 diabetes during adulthood [54,55]. In fact, the onset of diabetes depends on the ability of β-cells to respond to the increased demand for insulin that results from insulin resistance, with β-cells failure causing type-2 diabetes. 

The indexes of lipids metabolism were also altered in the offspring exposed to maternal malnutrition during the entire pregnancy, either by deficiency or excess. These animals show higher plasma levels of triglycerides and cholesterol and evidence changes in the relative amounts of HDL-c and LDL-c when compared to control individuals. At adulthood, the pigs exposed to both maternal under- and overnutrition during the entire pregnancy evidence *dyslipidemia*, a fourth symptom of the metabolic syndrome. This finding is even more concerning that the previous, since hypertriglyceridemia and dyslipidemia are morbidities usually found at more advances ages [56]. Hypertriglyceridemia is related primarily to the amount of visceral fat, significantly higher in the prenatally programmed piglets of our study, and, like in our study, may be indicative of impaired glucose tolerance [57-59]. In fact, the simultaneous increases in insulin resistance and plasma triglycerides in the current study support previous evidences of a link between insulin resistance and elevated triglycerides levels in blood and tissues [60-63].

Analysis of tissues, of fat composition, at adulthood also showed significant effects of prenatal nutrition. The three groups of pigs from under- and overfed pregnancies showed a higher fat desaturation than the control offspring; mainly, pigs from sows under- or overfed during the entire pregnancy, which have a higher proportion of monounsaturated fatty acids and, especially, of palmitoleic acid. Altered fatty acid metabolism has been implicated in the development of obesity and, moreover, the determination of the desaturation index is currently being studied as a potential biomarker of metabolic risk [64-66]. The desaturation index correlates with the activity of fatty acids desaturases; mainly with the stearoyl-CoA desaturase enzyme 1 (SCD1), the enzyme that catalyses the conversion of saturated to monounsaturated fatty acids. Increased SCD1 activity has been demonstrated in individuals with obesity and metabolic disorders, indicating enhanced lipogenesis [62-69]. Specifically, a low content of stearic acid and/or a high content in palmitoleic acid (precursor and product of SCD1 activity, respectively), like in the pigs exposed to maternal malnutrition in the current study, are considered independent and reliable markers of abdominal adiposity, triglyceridemia and alterations in insulin regulation [67,70]. 

## Conclusions

The results obtained in the present study indicate that exposure of Iberian pigs to maternal under- or overnutrition during pregnancy makes the offspring more prone to higher postnatal corpulence and adiposity. Such findings are supporting previous evidences found in the study of the DOHaD phenomena.

However, the first main finding in the current study is that a postnatal exposure to food abundance was not necessary for triggering effects on growth patterns and fatness. Changes in body development and adiposity were observed even in case of controlled intake during the first months after birth and even at early postnatal stages, during lactation. Afterwards, the exposure of these individuals to obesogenic diets increases significantly fat accumulation when compared to offspring from pregnancies with balanced diets. 

The second main finding of the current study is the fact that these effects can be amplified in the female sex. The effects of sex are even more remarkable in offspring exposed to maternal undernutrition during the last two thirds of pregnancy. In this case, patterns of body growth and corpulence are similar to control piglets but females have a strong predisposition to obesity, and even stronger after exposition to obesogenic diets. 

Finally, such alterations in patterns of growth and fattening could be related to the development of metabolic syndrome (central obesity, impaired glucose regulation, insulin resistance and, dyslipidemia) and prodrome of type-2 diabetes at very early life stages.

These findings provide a basis for clarifying the interaction between genetic and environmental factors in the determination of adult phenotype. The distinctive hallmark of our study is the use of the Iberian pig as a large animal model genetically adapted, for generations, to harsh environments and food scarcity. Hence, the data obtained are unique in translational studies of obesity and associated disorders in developing countries like China, India and Middle East countries, in which populations adapted for surviving in harsh environments are currently exposed to nutrient excess. 

## Supporting Information

Table S1 **Effects of sex and maternal nutrition on offspring body growth.** Changes over time in mean values for body weight, body volume and Body Mass Indexes BMI1 and BMI2 in male and female Iberian piglets born from sows fed, during the entire pregnancy, with a diet fulfilling either 100% (CONTROL), or 160% (OVERFED) or 50% of daily maintenance requirements for gestation (UNDERFED. A fourth group (LATE-UNDERFED) was born from females fed with 100% maintenance requirements until Day 35 of pregnancy, like the CONTROL group, but restricted to 50% of such amount from Day 36 onwards, like the UNDERFED group.
(DOCX)

Table S2 **Effects of sex and maternal nutrition on offspring adiposity.** Changes over time in mean values for subcutaneous back-fat depth, determined by ultrasonography and MRI, and area of visceral fat depot, at the level of the third lumbar vertebra, in male and female Iberian piglets born from sows fed, during the entire pregnancy, with a diet fulfilling either 100% (CONTROL), or 160% (OVERFED) or 50% of daily maintenance requirements for gestation (UNDERFED. A fourth group (LATE-UNDERFED) was born from females fed with 100% maintenance requirements until Day 35 of pregnancy, like the CONTROL group, but restricted to 50% of such amount from Day 36 onwards, like the UNDERFED group.
(DOCX)

Table S3 **Effects of sex and maternal nutrition on leptin secretion.** Changes over time in mean values for plasma leptin concentrations (ng/ml)in male and female Iberian piglets born from sows fed, during the entire pregnancy, with a diet fulfilling either 100% (CONTROL), or 160% (OVERFED) or 50% of daily maintenance requirements for gestation (UNDERFED. A fourth group (LATE-UNDERFED) was born from females fed with 100% maintenance requirements until Day 35 of pregnancy, like the CONTROL group, but restricted to 50% of such amount from Day 36 onwards, like the UNDERFED group.
(DOCX)
