# Supplementary material for: Maternal Malnutrition and Offspring Sex Determine Juvenile Obesity and Metabolic Disorders in a Swine Model of Leptin Resistance
Source: PLoS One. 2013 Oct 24;8(10):e78424. doi: 10.1371/journal.pone.0078424 (PMC3813450; doi:10.1371/journal.pone.0078424)
Supplement: Table S1 — Effects of sex and maternal nutrition on offspring body growth. Changes over time in mean values for body weight, body volume and Body Mass Indexes BMI1 and BMI2 in male and female Iberian piglets born from sows fed, during the entire pregnancy, with a diet fulfilling either 100% (CONTROL), or 160% (OVERFED) or 50% of daily maintenance requirements for gestation (UNDERFED. A fourth group (LATE-UNDERFED) was born from females fed with 100% maintenance requirements until Day 35 of pregnancy, like the CONTROL group, but restricted to 50% of such amount from Day 36 onwards, like the UNDERFED group. (DOCX) [file pone.0078424.s001.docx]

**Supplementary Table 1. Effects of sex and maternal nutrition on offspring body growth**. Changes over time in mean values for body weight, body volume and Body Mass Indexes BMI1 and BMI2 in male and female Iberian piglets born from sows fed, during the entire pregnancy, with a diet fulfilling either 100% (CONTROL), or 160% (OVERFED) or 50% of daily maintenance requirements for gestation (UNDERFED. A fourth group (LATE-UNDERFED) was born from females fed with 100% maintenance requirements until Day 35 of pregnancy, like the CONTROL group, but restricted to 50% of such amount from Day 36 onwards, like the UNDERFED group.

|  | | **CONTROL** | | **OVERFED** | | **UNDERFED** | | **LATE-UNDERFED** | |
| --- | --- | --- | --- | --- | --- | --- | --- | --- | --- |
|  | **Days of age** | **FEMALE** | **MALE** | **FEMALE** | **MALE** | **FEMALE** | **MALE** | **FEMALE** | **MALE** |
| **Body-weight (Kg)** | **0** | 1.3±0.1 | 1.3±0.1 | 1.3±0.2 | 1.6±0.2 | 1.5±0.2 | 1.4±0.2 | 1.3±0.2 | 1.4±0.2 |
|  | **28** | 8.2±0.4 | 8.9±0.2 | 6.7±0.6 | 7.7±0.5 | 7.0±0.4 | 7.6±0.3 | 8.3±0.3 | 7.8±0.7 |
|  | **60** | 13.4±0.3 | 15.9±0.6 | 12.2±0.9 | 13.5±0.5 | 13.6±0.9 | 15.2±0.4 | 15.4±0.5 | 13.4±1.1 |
|  | **90** | 20.7±0.7 | 24.1±0.7 | 29.8±1.5 | 32.9±0.8 | 29.6±1.8 | 34.9±1.1 | 22.9±1.2 | 22.0±1.4 |
|  | **120** | 28.2±0.7 | 31.7±1.1 | 43.9±2.1 | 48.3±0.9 | 44.1±2.3 | 50.3±1.3 | 32.2±1.6 | 29.1±1.7 |
|  | **150** | 38.9±0.7 | 43.0±1.2 | 66.3±3.1 | 68.7±2.0 | 67.7±1.8 | 70.2±1.4 | 43.6±1.6 | 39.3±1.6 |
|  | **180** | 52.0±1.1 | 58.0±1.2 | 97.5±2.9 | 101.7±2.3 | 99.1±2.4 | 96.9±2.3 | 56.1±2.5 | 51.6±1.9 |
|  | **210** | 64.8±1.4 | 73.2±1.9 | 118.3±3.4 | 122.9±4.0 | 116.1±3.0 | 112.0±2.2 | 74.2±3.7 | 66.5±2.4 |
|  | **240** | 88.7±1.6 | 97.9±2.0 | 125.2±4.2 | 134.3±3.3 | 129.9±3.1 | 124.8±2.4 | 98.1±4.8 | 93.8±2.5 |
| **Volume (cm^3^)** | **0** | 732.7±18.4 | 739.4±19.1 | 655.4±13.3 | 743.1±17.2 | 739.0±22.2 | 704.4±19.6 | 696.0±17.3 | 682.3±21.2 |
|  | **28** | 4153.5±109.3 | 4320.0±211.3 | 2855.5±169.3 | 3162.5±201.3 | 3028.1±188.9 | 3238.1±231.9 | 4094.8±123.6 | 3809.5±111.3 |
|  | **60** | 6966.3±384.9 | 8050.0±401.3 | 7442.5±396.5 | 8683.4±506.3 | 7879.7±423.6 | 8696.3±639.3 | 7359.9±511.2 | 6264.8±326.9 |
|  | **90** | 11597.1±729.6 | 13158.4±835.2 | 14891.8±963.9 | 15892.2±984.3 | 14917.7±639.1 | 16600.0±724.6 | 12891.2±734.6 | 11377.1±684.9 |
|  | **120** | 22968.4±983.9 | 27792.4±1171.9 | 34866.0±1673.4 | 37871.7±1254.9 | 27599.6±1431.6 | 24733.2±1645.7 | 37829.2±1476.1 | 40018.1±1221.3 |
|  | **150** | 34027.1±1168.1 | 37076.8±877.7 | 63090.1±3281.0 | 64443.1±2469.7 | 39667.9±1171.9 | 33162.9±1736.7 | 62974.6±1828.5 | 66847.6±1846.3 |
|  | **180** | 55251.3±1942.1 | 63965.4±1747.9 | 95949.4±3534.1 | 105839.1±3708.8 | 63917.9±2940.8 | 57736.5±2375.9 | 98867.7±3407.8 | 99419.8±3918.1 |
|  | **210** | 69723.6±1400.3 | 82208.8±1907.2 | 115078.6±5553.9 | 130440.3±5260.5 | 83056.1±3883.9 | 72304.6±2211.1 | 119341.8±4497.9 | 122213.2±2633.2 |
|  | **240** | 89246.4±1401.3 | 103906.0±2542.8 | 123718.7±5378.9 | 150645.1±6769.6 | 103347.0±4736.5 | 97652.3±2743.0 | 137799.1±4286.6 | 132460.5±3417.6 |

| **BMI1** | **0** | 3.5±0.2 | 3.6±0.2 | 3.8±0.3 | 4.1±0.2 | 4.0±0.3 | 3.8±0.3 | 3.5±0.3 | 3.5±0.3 |
| --- | --- | --- | --- | --- | --- | --- | --- | --- | --- |
|  | **28** | 12.4±0.7 | 13.4±0.8 | 11.4±0.7 | 12.8±0.7 | 11.6±0.6 | 12.6±0.8 | 12.1±0.9 | 11.7±0.7 |
|  | **60** | 16.5±0.9 | 19.5±1.0 | 16.2±1.0 | 17.5±0.9 | 17.8±1.1 | 19.3±1.2 | 18.4±1.1 | 17.6±1.2 |
|  | **90** | 20.3±1.1 | 23.0±1.1 | 25.8±1.2 | 28.2±1.1 | 25.3±0.9 | 30.21.1± | 21.0±1.1 | 21.1±1.2 |
|  | **120** | 40.2±1.4 | 42.4±1.2 | 55.1±2.2 | 61.4±1.5 | 54.3±3.0 | 61.9±1.4 | 42.5±1.8 | 40.2±1.4 |
|  | **150** | 49.3±1.3 | 50.8±1.5 | 59.3±2.1 | 61.8±1.2 | 61.9±1.4 | 63.2±1.5 | 50.6±1.9 | 49.1±1.1 |
|  | **180** | 51.0±1.0 | 55.4±0.6 | 84.5±1.9 | 87.2±2.2 | 84.7±1.8 | 83.8±3.0 | 51.5±1.3 | 49.5±1.2 |
|  | **210** | 57.5±1.1 | 62.4±1.3 | 99.2±1.9 | 98.6±2.3 | 94.0±2.0 | 88.1±2.0 | 62.1±2.3 | 58.7±1.9 |
|  | **240** | 75.3±1.6 | 79.6±1.3 | 100.3±3.1 | 100.4±1.8 | 98.0±2.8 | 96.3±2.7 | 78.2±2.7 | 77.8±1.3 |

| **BMI2** | **0** | 19.0±0. 1 | 19.4±0. 1 | 20.5±0. 1 | 21.0±0. 1 | 19.2±0.1 | 20.3±0.1 | 20.3±0. 1 | 20.2±0.1 |
| --- | --- | --- | --- | --- | --- | --- | --- | --- | --- |
|  | **28** | 20.1±0.4 | 20.7±0.5 | 23.5±0.3 | 24.3±0.6 | 23.6±0.5 | 23.5±0.4 | 20.3±0.3 | 20.8±0.3 |
|  | **60** | 19.4±0.3 | 20.0±0.4 | 20.4±0.4 | 20.5±0.3 | 20.4±0.5 | 20.6±0.4 | 20.9±0.4 | 21.4±0.3 |
|  | **90** | 18.0±0.2 | 18.4±0.3 | 20.1±0.4 | 20.8±0.3 | 20.0±0.2 | 21.00.3 | 17.7±0.2 | 19.4±0.4 |
|  | **120** | 12.4±0.6 | 11.5±0.3 | 12.7±0.4 | 12.9±0.5 | 11.7±0.4 | 12.6±0.2 | 11.7±0.2 | 11.9±0.3 |
|  | **150** | 11.5±0.4 | 11.7±0.4 | 10.6±0.3 | 10.7±0.3 | 10.8±0.1 | 10.5±0.3 | 11.0±0.2 | 11.9±0.3 |
|  | **180** | 9.5±0.3 | 9.1±0.2 | 10.2±0.2 | 9.7±0.2 | 10.1±0.2 | 9.9±0.4 | 8.8±0.1 | 9.0±0.2 |
|  | **210** | 9.3±0.2 | 8.9±0.2 | 10.4±0.3 | 9.5±0.2 | 9.8±0.2 | 9.3±0.2 | 9.0±0.3 | 9.2±0.1 |
|  | **240** | 10.0±0.1 | 9.4±0.1 | 10.2±0.3 | 9.0±0.2 | 9.5±0.3 | 9.6±0.3 | 9.5±0.1 | 9.6±0.1 |
