# Supplementary material for: Maternal Malnutrition and Offspring Sex Determine Juvenile Obesity and Metabolic Disorders in a Swine Model of Leptin Resistance
Source: PLoS One. 2013 Oct 24;8(10):e78424. doi: 10.1371/journal.pone.0078424 (PMC3813450; doi:10.1371/journal.pone.0078424)
Supplement: Table S2 — Effects of sex and maternal nutrition on offspring adiposity. Changes over time in mean values for subcutaneous back-fat depth, determined by ultrasonography and MRI, and area of visceral fat depot, at the level of the third lumbar vertebra, in male and female Iberian piglets born from sows fed, during the entire pregnancy, with a diet fulfilling either 100% (CONTROL), or 160% (OVERFED) or 50% of daily maintenance requirements for gestation (UNDERFED. A fourth group (LATE-UNDERFED) was born from females fed with 100% maintenance requirements until Day 35 of pregnancy, like the CONTROL group, but restricted to 50% of such amount from Day 36 onwards, like the UNDERFED group. (DOCX) [file pone.0078424.s002.docx]

**Supplementary Table 2. Effects of sex and maternal nutrition on offspring adiposity.** Changes over time in mean values for subcutaneous back-fat depth, determined by ultrasonography and MRI, and area of visceral fat depot, at the level of the third lumbar vertebra, in male and female Iberian piglets born from sows fed, during the entire pregnancy, with a diet fulfilling either 100% (CONTROL), or 160% (OVERFED) or 50% of daily maintenance requirements for gestation (UNDERFED. A fourth group (LATE-UNDERFED) was born from females fed with 100% maintenance requirements until Day 35 of pregnancy, like the CONTROL group, but restricted to 50% of such amount from Day 36 onwards, like the UNDERFED group.

|  | | **CONTROL** | | **OVERFED** | | **UNDERFED** | | **LATE-UNDERFED** | |
| --- | --- | --- | --- | --- | --- | --- | --- | --- | --- |
|  | **Days of age** | **FEMALE** | **MALE** | **FEMALE** | **MALE** | **FEMALE** | **MALE** | **FEMALE** | **MALE** |
| **Subcutaneous back-fat depth (mm, US)** | **60** | 5.3±0.5 | 5.9±0.4 | 8.9±0.5 | 9.6±0.3 | 9.4±0.3 | 9.3±0.4 | 5.5±0.5 | 4.8±0.5 |
|  | **90** | 5.9±0.3 | 7.2±0.4 | 12.4±0.9 | 13.5±0.6 | 14.0±0.5 | 14.2±0.5 | 6.3±0.3 | 5.6±0.5 |
|  | **120** | 7.3±0.6 | 8.0±0.5 | 16.2±1.0 | 17.4±0.7 | 15.9±0.9 | 17.9±1.1 | 7.9±0.7 | 7.7±0.7 |
|  | **150** | 10.5±0.8 | 11.8±0.6 | 21.1±1.1 | 24.4±0.6 | 21.2±0.7 | 23.0±1.0 | 11.2±0.6 | 10.3±0.5 |
|  | **180** | 14.4±1.1 | 19.0±1.0 | 32.0±1.4 | 35.4±1.5 | 32.2±1.2 | 35.9±1.0 | 18.5±0.9 | 18.1±1.1 |
|  | **210** | 17.0±1.1 | 21.8±1.1 | 38.0±1.0 | 40.3±1.2 | 37.5±1.2 | 38.5±0.8 | 21.4±1.2 | 19.7±0.9 |
|  | **240** | 27.5±1.7 | 31.1±1.3 | 42.5±1.3 | 43.6±1.9 | 41.4±2.1 | 40.0±1.4 | 30.6±1.5 | 31.1±1.2 |

| **Subcutaneous back-fat depth (mm, MRI)** | **120** | 17.9±1.9 | 15.8±2.5 | 27.9±2.1 | 28.3±2.2 | 26.5±6.6 | 32.6±2.3 | 16.4±2.7 | 13.4±2.4 |
| --- | --- | --- | --- | --- | --- | --- | --- | --- | --- |
|  | **180** | 29.0±2.1 | 26.7±2.1 | 59.1±1.6 | 63.2±5.8 | 57.0±3.6 | 61.0±2.1 | 25.5±1.3 | 28.7±3.9 |
| **Visceral fat depot**  **(mm^2^, MRI)** | **120** | 321.2±36.0 | 550.2±90.9 | 893.8±65.1 | 1150.0±129.9 | 1283.8±292.6 | 917.4±53.1 | 423.5±113.8 | 285.3±73.9 |
|  | **180** | 946.6±84.4 | 1130.7±166.5 | 2714.3±214.9 | 3001.8±341.1 | 2559.2±178.3 | 2614.3±296.7 | 1384.2±173.6 | 915.0±154.3 |
