# Supplementary material for: Maternal Malnutrition and Offspring Sex Determine Juvenile Obesity and Metabolic Disorders in a Swine Model of Leptin Resistance
Source: PLoS One. 2013 Oct 24;8(10):e78424. doi: 10.1371/journal.pone.0078424 (PMC3813450; doi:10.1371/journal.pone.0078424)
Supplement: Table S3 — Effects of sex and maternal nutrition on leptin secretion. Changes over time in mean values for plasma leptin concentrations (ng/ml)in male and female Iberian piglets born from sows fed, during the entire pregnancy, with a diet fulfilling either 100% (CONTROL), or 160% (OVERFED) or 50% of daily maintenance requirements for gestation (UNDERFED. A fourth group (LATE-UNDERFED) was born from females fed with 100% maintenance requirements until Day 35 of pregnancy, like the CONTROL group, but restricted to 50% of such amount from Day 36 onwards, like the UNDERFED group. (DOCX) [file pone.0078424.s003.docx]

**Supplementary Table 3. Effects of sex and maternal nutrition on leptin secretion.** Changes over time in mean values for plasma leptin concentrations (ng/ml)in male and female Iberian piglets born from sows fed, during the entire pregnancy, with a diet fulfilling either 100% (CONTROL), or 160% (OVERFED) or 50% of daily maintenance requirements for gestation (UNDERFED. A fourth group (LATE-UNDERFED) was born from females fed with 100% maintenance requirements until Day 35 of pregnancy, like the CONTROL group, but restricted to 50% of such amount from Day 36 onwards, like the UNDERFED group.

|  | | **CONTROL** | | **OVERFED** | | **UNDERFED** | | **LATE-UNDERFED** | |
| --- | --- | --- | --- | --- | --- | --- | --- | --- | --- |
|  | **Days of age** | **FEMALE** | **MALE** | **FEMALE** | **MALE** | **FEMALE** | **MALE** | **FEMALE** | **MALE** |
| **Leptin (ng/ml)** | **120** | 3.8±0.8 | 4.3±0.7 | 6.1±0.5 | 6.7±0.6 | 6.0±0.8 | 6.7±0.5 | 4.4±0.4 | 4.8±0.9 |
|  | **180** | 9.3±1.8 | 5.8±0.6 | 10.7±1.0 | 6.3±0.6 | 10.5±0.5 | 6.5±0.5 | 4.0±0.3 | 7.1±0.7 |
